# Factors Associated with Deworming Medication Utilization among Pregnant Women in Benin: Evidence from the Demographic and Health Survey

**DOI:** 10.3390/tropicalmed8030166

**Published:** 2023-03-12

**Authors:** Daniel Amoak, Satveer Dhillon, Roger Antabe, Yujiro Sano, Isaac Luginaah

**Affiliations:** 1 Department of Geography and Environment, Western University, London, ON N6A 5C2, Canada; 2 Department of Health and Society, University of Toronto Scarborough, Scarborough, ON M1C 1A4, Canada; 3 Department of Sociology and Anthropology, Nipissing University, North Bay, ON P1B 8L7, Canada

**Keywords:** deworming medication, soil-transmitted helminths, pregnant women, Benin, demographic and health survey, neglected tropical diseases

## Abstract

Deworming medication utilization is a useful strategy to reduce the burden of anemia among pregnant women. Yet, we know very little about the prevalence and correlates of deworming medication utilization among pregnant women in sub-Saharan Africa, including Benin. To address this void in the literature, we used the 2017–2018 Benin Demographic and Health Survey and applied logistic regression analysis to explore the demographic, socioeconomic, and healthcare factors associated with deworming medication utilization in Benin. We found that deworming medication coverage was 65% at the national level. We observed that women aged 35–49 years were less likely to use deworming medication compared to those aged 15–24 years (OR = 0.79, *p* < 0.01). Compared to Christian women, Muslim women (OR = 0.70, *p* < 0.01) and women of other religions (OR = 0.51, *p* < 0.01) were also less likely to use deworming medication. Moreover, women with lower levels of education and household wealth, as well as unemployed women, were less likely to use deworming medication in comparison to their educated, richer, and employed counterparts. Women who visited ANC fewer than eight times were also less likely to use deworming medication compared to their counterparts who did so eight times or more (OR = 0.65, *p* < 0.001). Based on these findings, we discussed several implications for policymakers.

## 1. Introduction

Intestinal parasitic infections remain a major health challenge in the Global South [1,2,3]. The World Health Organization (WHO) reported that about 688 million adolescent girls and women of reproductive age reside in areas with intestinal worm endemicity [1,4]. Pregnant women, particularly those residing in sub-Saharan Africa (SSA), are at an increased risk of intestinal worm infestations, such as hookworm, Giardia intestinalis, Entamoeba histolytica/dispar/moshkovskii, Enterobius vermicularis, Hymenolepis nana, Ascaris lumbricoides, Trichuris trichiura, and whipworm [1,5,6]. Being infected by an intestinal worm can cause internal bleeding, intestinal obstruction, and inflammation, along with reducing the availability of nutrients [7]. These factors contribute to iron deficiency anemia, which is especially harmful to pregnant women as they have higher requirements of iron [1,7]. Along with impacting pregnant women’s health and nutrition, anemia can negatively impact newborn health due to decreased levels of hemoglobin [8]. Anemia increases the risk of stunting, wasting, infant mortality and morbidity, and increases the risk of low birth weight [1]. In regions with high maternal and infant mortality rates, including Benin, eradicating intestinal worm infestation is crucial for achieving Sustainable Development Goal (SDG) 3.1, which targets improving the health of mothers and infants [9].

Exposure to intestinal worms is driven primarily by poor access to improved water, sanitation, and hygiene [8,10]. Recent estimates reveal that about 73% of people residing in Benin have no access to a handwashing facility, 58% of households practice open defecation and 90% live in communities where at least one household practices open defecation [11]. These unhygienic practices expose many households to adverse health impacts, including intestinal parasitic infections [12]. To reduce parasitic intestinal infections, the WHO Neglected Tropical Disease (NTD) Roadmap and London declaration focused on accelerating progress towards eliminating specific NTDs. As a result of these efforts, the DeWorm3 Project was established to assess the feasibility of using mass drug administration to interrupt the transmission of soil-transmitted helminths (STHs), using study sites in Benin, along with India and Malawi [13]. The success of this intervention hinged on both high levels of treatment coverage and high levels of compliance [14]. While progress has been made through mass drug administration campaigns of donated medicines, these campaigns targeted and prioritized children in endemic regions over women of reproductive age [15]. As such, only 23% of pregnant women received deworming treatment [16].

In Benin, previous studies have found that the prevalence of STHs among at-risk populations ranges from 5% to 60% [17,18,19]. Among children of school-going age (8–14 years), for instance, a national study highlighted the presence of hookworm in all 30 districts surveyed, with the prevalence ranging from 1.2% to 60% [18]. Similarly, a study in the Comé district also found children to be at an increased risk of STH, with an overall prevalence of 5.3% [19]. Specifically, a prospective cohort study conducted amongst pregnant women in the Allada District found that the prevalence of intestinal worms among pregnant women during their first antenatal care (ANC) visit was as high as 11.5% [17]. This high prevalence is concerning, as the WHO recommends a prevalence rate of below 1% for optimal public health.

Reflecting on these high estimates, the Ministry of Health in Benin has focused on developing strategies to control NTDs, such as STHs, among at-risk groups, including pregnant women [19]. Despite these efforts, research shows that there are unique barriers to proper hygienic practices in Benin, such as lack of handwashing and toilet facilities, which have been documented as significant predictors of public health threats, including intestinal parasitic infections [17]. In addition to proper hygienic practices, deworming medication is a crucial tool for treating intestinal parasitic infections to reduce STH-related morbidity among high-risk populations, including pregnant women [20]. Specifically, the WHO recommends providing pregnant women with a single dose of albendazole or mebendazole after their first trimester of pregnancy in areas such as Benin, where the prevalence of worm infections is high [4]. Furthermore, by reducing birth complications, deworming has been shown to protect the unborn baby against neonatal mortality and low birthweight [20,21,22]. For example, receiving deworming medicine during pregnancy can reduce neonatal mortality by 14% and decrease the odds of low birth weight by 11% [20]. Research also shows that it can reduce maternal anemia [22].

Although deworming has positive health benefits, several barriers can impact the utilization of deworming medication for pregnant women. For one, while no severe side effects have been reported, pregnant women have complained about minor side effects, such as vomiting, nausea, and diarrhea [23]. These side effects may discourage pregnant women from using deworming medications, which highlights the importance of informing factual knowledge about deworming medication to expectant mothers. In addition, the lack of affordability with poor access to deworming medication has been identified as a major barrier in Benin [11]. For example, a multi-country analysis in SSA concluded that women with higher levels of household wealth were also more likely to use deworming medication [5]. Another study focusing on Tanzania highlighted that urban-dwelling women were more likely to use deworming medication compared to those in rural areas [7]. Studies also showed that those who are older are more likely to use deworming medication in SSA [5]. Along with socioeconomic barriers to deworming utilization, it was also reported that geographic disparities in the concentration of health facilities favour some regions in Benin [24]. In this context, poor access to healthcare centers may pose as a barrier to accessing deworming medication, especially in rural areas where healthcare resources are rather restricted.

Despite the high risk of being exposed to intestinal worms in SSA, the literature pays very little attention to the prevalence and correlates of deworming medication utilization among pregnant women in Benin. To address this void in the literature, this study used a nationally representative survey to explore the demographic, socioeconomic, and healthcare factors associated with deworming medication utilization in Benin. The findings from this study could help policymakers inform policies regarding national deworming medication amongst pregnant women in Benin.

## 2. Materials and Methods

### 2.1. Data

We used the 2017–18 Benin Demographic and Health Survey (BDHS), which was collected by the National Institute of Statistics and Economic Analysis, under the supervision of the Ministry of Planning and Development, with technical assistance of the ICF International through the USAID-funded DHS program. The BDHS is suitable for this study, with high-quality and reliable information on basic demographic indices and health indicators, including the use of deworming medication. The BDHS employed a two-stage sampling framework, in which a stratified probability proportional to size sampling method was applied, and conducted face-to-face interviews with 15,928 women and 7595 men, with a response rate of 98% for both women and men. For the purpose of this study, we focused on 8944 women who have given birth in the last five years and answered questions about the use of deworming medication.

### 2.2. Measures

We created the dependent variable called ‘use of deworming medication’, where women were coded as ‘yes’ when they used deworming medications during the last pregnancy (0 = no; 1 = yes). Informed by previous research [5], we introduced three sets of the independent variables, namely demographic, socioeconomic, and health care variables. Demographic variables included age of respondents (0 = 15–24; 1 = 25–34; 2 = 35–49), marital status (0 = married; 1 = not married), religion (0 = Christian; 1 = Muslim; 2 = traditionalist; 3 = other; 4 = no religion), region of residence (0 = Ouémé; 1 = Alibori; 2 = Atacora; 3 = Atlantic; 4 = Borgou; 5 = Collines; 6 = Couffo; 7 = Donga; 8 = Littoral; 9 = Mono; 10 = Plateau; 11 = Zou), and place of residence (0 = urban; 1 = rural). There were three socioeconomic variables including education (0 = secondary/higher education; 1 = primary education; 2 = no education), household wealth quintile (0 = richest; 1 = richer; 2 = middle; 3 = poorer; 4 = poorest), and employment status (0 = employed; 1 = unemployed). Finally, we introduced a number of ANC visits as a health care variable (0 = eight times or more; 1 = fewer than eight times).

### 2.3. Data Analysis

We employed three different analyses for this study. First, we used univariate analysis to describe the characteristics of our analytical sample. Second, bivariate analysis was conducted to understand the unadjusted associations between the dependent and independent variables. Third, we relied on multivariate analysis to simultaneously include the independent variables to produce net estimates. For bivariate and multivariate analysis, we used logistic regression analysis due to the dichotomous nature of the dependent variable [25,26]. Results were shown with odds ratios (ORs). ORs larger than 1 indicated that women were more likely to use deworming medication, while those smaller than 1 pointed to lower odds of using them. All analyses were carried out using STATA 17 (State Corp, College Station, TX, USA). The ‘svy’ function is applied in statistical analysis to adjust for the cluster sampling design, as well as sampling weights.

## 3. Results

Table 1 shows sample characteristics. We found that 65% of women used deworming medication during their last pregnancy. In terms of demographic factors, it was observed that the largest age group was 25–34 (49%), followed by 35–49 (27%) and 15–24 (24%). We also found that 50%, 33%, and 10% of women were Christian, Muslim, and traditionalist, respectively. The largest proportion of women lived in Alibori (13%) and Borgou (13%), followed by Atlantic (12%) and Zou (10%). More women lived in rural areas (61%) compared to urban areas (39%). For socioeconomic characteristics, it was also found that about two thirds of women (65%) did not have any formal education, although 81% were employed. The majority (91%) also did not visit ANC eight times or more.

Table 2 shows bivariate and multivariate findings. At the bivariate level, we found that a range of demographic, socioeconomic, and healthcare factors were significantly associated with the use of deworming medication. For demographic factors, we observed that women aged 35–49 were less likely to use deworming medication compared to those aged 15–24 (OR = 0.76, *p* < 0.001). Compared to Christian women, Muslim (OR = 0.31, *p* < 0.001) and traditional women (OR = 0.61, *p* < 0.001), as well as women with other (OR = 0.63, *p* < 0.05) and no religion (OR = 0.29, *p* < 0.001), were also less likely to use deworming medication. The region of residence was also associated with the use of deworming medication, indicating that women in Alibori (OR = 0.06, *p* < 0.001), Atacora (OR = 0.03, *p* < 0.001), Atlantic (OR = 0.53, *p* < 0.01), Borgou (OR = 0.03, *p* < 0.001), Collines (OR = 0.12, *p* < 0.001), Couffo (OR = 0.09, *p* < 0.001), Donga (OR = 0.12, *p* < 0.001), Mono (OR = 0.17, *p* < 0.001), Plateau (OR = 0.20, *p* < 0.001), and Zou (OR = 0.23, *p* < 0.001) were less likely to use deworming medication compared to women in Ouémé. Similarly, women in rural areas were less likely to use deworming medication than urban women (OR = 0.55, *p* < 0.001). For socioeconomic factors, it was found that women with primary (OR = 0.63, *p* < 0.001) and no education (OR = 0.31, *p* < 0.001) were less likely to use deworming medication in comparison to women with secondary/higher education. Furthermore, women whose household wealth belonged to the ‘richer’ (OR = 0.41, *p* < 0.001), ‘middle’ (OR = 0.25, *p* < 0.001), ‘poorer’ (OR = 0.19, *p* < 0.001), and ‘poorest’ (OR = 0.10, *p* < 0.001) category were less likely to use deworming medication compared to their ‘richest’ counterparts. Compared to employed women, unemployed women were also less likely to use deworming medication (OR = 0.51, *p* < 0.001). For health care factors, it was found that women who visited ANC fewer than eight times were less likely to use deworming medication compared to their counterparts, who did so eight times or more (OR = 0.26, *p* < 0.001).

Multivariate results are largely consistent with the bivariate results, except the relationship between deworming medication use and the place of residence, not being statistically significant. For example, we observed that women aged 35–49 were less likely to use deworming medication compared to those aged 15–24 (OR = 0.79, *p* < 0.01). Compared to Christian women, Muslim women (OR = 0.70, *p* < 0.01) and women with other religion (OR = 0.51, *p* < 0.01) were also less likely to use deworming medication. The region of residence was also associated with the use of deworming medication, indicating that women in Alibori (OR = 0.16, *p* < 0.001), Atacora (OR = 0.06, *p* < 0.001), Atlantic (OR = 0.52, *p* < 0.01), Borgou (OR = 0.06, *p* < 0.001), Collines (OR = 0.15, *p* < 0.001), Couffo (OR = 0.10, *p* < 0.001), Donga (OR = 0.22, *p* < 0.001), Littoral (OR = 0.34, *p* < 0.01), Mono (OR = 0.19, *p* < 0.001), Plateau (OR = 0.28, *p* < 0.001), and Zou (OR = 0.25, *p* < 0.001) were less likely to use deworming medication compared to women in Ouémé. In terms of socioeconomic status, it was found that women with primary (OR = 0.61, *p* < 0.001) and no education (OR = 0.49, *p* < 0.001) were less likely to use deworming medication in comparison to women with secondary/higher education. Furthermore, women whose household wealth belonged to the ‘richer’ (OR = 0.64, *p* < 0.001), ‘middle’ (OR = 0.52, *p* < 0.001), ‘poorer’ (OR = 0.46, *p* < 0.001), and ‘poorest’ (OR = 0.31, *p* < 0.001) category were less likely to use deworming medication compared to their ‘richest’ counterparts. Compared to employed women, unemployed women were also less likely to use deworming medication (OR = 0.53, *p* < 0.001). For health care factors, it was found that women who visited ANC fewer than eight times were less likely to use deworming medication compared to their counterparts, who did so eight times or more (OR = 0.65, *p* < 0.001).

## 4. Discussion

In this study, we analyzed the factors associated with deworming medication use among pregnant women in Benin. We found that the uptake of deworming medication remains low in Benin, with a third (35%) of pregnant women not using deworming medication. The low usage of deworming medication may be a barrier to achieving SDG targets for maternal and infant health, and there is a need for targeting interventions to improve the use of deworming medication. Our analysis further points to the complex interplay of demographic, socioeconomic, geographic and health factors that impact the uptake of deworming medication in Benin.

We found that pregnant women from the oldest age cohort (i.e., 35–49) were less likely to use deworming medication than their youngest counterparts (i.e., 15–24). While this finding may contrast with that of the existing literature [5], we provided some plausible explanations for our findings. First, in the context of Benin, where there is an increased maternal policy attention on especially younger expectant mothers, they may have better opportunities to be informed or access deworming medication relative to their older counterparts [27]. Further, it is possible that younger women in Benin may have a heightened risk perception of pregnancy complications, compelling them to increase their interaction with healthcare spaces where they can access deworming services [28]. The WHO also asserts that adolescents and young women may often have received deworming medication at their preschool and school years, and are thus familiar with the health benefits of taking deworming medication compared to older adults [1]. Conversely, older women, often with higher parity (more previous pregnancies), may have more knowledge and experience from their previous pregnancies and deliveries and may develop the confidence to perceive that healthcare use, including deworming treatment, is not as necessary [29,30]. Furthermore, a higher birth order implies a larger family size and therefore, lower resources, such as money, may be available to access healthcare services, such as deworming medication [29]. This finding is consistent with a previous study in Ghana, asserting that women’s use of health services, such as deworming treatment, reduces as they grow older [31].

Additionally, our study revealed that religious affiliations were associated with deworming medication uptake among pregnant women. Compared to their Christian counterparts, Muslim women were found to be less likely to have received deworming medication. This finding is consistent with earlier studies in Benin and Nigeria, suggesting that Muslim mothers had the lowest motivation towards receiving vaccinations relative to their Christian counterparts [32,33]. In the highly religious context of West Africa, health and religion are often interconnected, as some illnesses are believed to have spiritual causes [34]. These religious beliefs can prevent some women from accessing recommended maternal healthcare services, such as deworming medication [35]. In addressing this challenge, it is critical for public health practitioners to work with religious leaders to increase women’s trust in medications and reduce misconceptions surrounding illnesses and their root causes to improve the uptake of deworming medication.

In addition, we also found that pregnant women living outside of the Ouémé region reported lower odds of using deworming medication during their pregnancy in comparison to women living in that region. This finding may be explained by other studies [24,36,37], which point to the geographic disparities in access to healthcare services in SSA. In analyzing a deworming program in Benin, Geyer et al. found that the overwhelming rural context of Comé served as a barrier to effective mass drug administration delivery, as most resources were concentrated in the urban areas [14]. Similarly, it has also been suggested that long distances to healthcare centers in many regions, such as Borgou, Collines, and Atakora, may explain the low uptake of deworming medication [24]. Research also shows that the lack of healthcare facilities in Northern Benin may have contributed to lower rates of healthcare access among women, which has implications for accessing deworming medication [38]. Based on findings from this research, as well as previous studies, it may be critical for policymakers to expand healthcare facilities in northern Benin, which may reduce the location-based inequality on the use of deworming medication among pregnant women.

Furthermore, our results highlighted that pregnant women with primary education and no formal education were less likely to use deworming medication than their counterparts with secondary education. Other studies also report that higher educational attainment is associated with deworming medication utilization in SSA [5,14]. It is possible that educated women may have greater knowledge about recommended maternal health practices, which may increase their perceived importance of receiving deworming medications during pregnancy [31]. Furthermore, women with lower education often hold misconceptions regarding medication, potentially creating an environment where they may be less likely to rely on deworming medication [39,40,41,42,43]. To address this disparity, it may be useful to establish community-level educational campaigns specifically targeting women with low educational attainment, with a focus on dispelling misconceptions surrounding deworming medication [14]. These educational campaigns can be implemented alongside existing platforms, such as antenatal clinics, educational institutions, and religious institutions [1,44].

We also found that women belonging to households with lower wealth were less likely to use deworming medication in comparison to their wealthier counterparts. This finding is consistent with other studies in SSA on healthcare utilization, including the uptake of deworming medication [45,46]. Studies have noted that lower-income households in Benin have decreased access to basic necessities, such as improved water, shelter, and food. As a result, available resources are likely to be used for meeting these basic needs rather than healthcare needs, such as deworming medication. Even in regions where deworming medication is freely available as part of ANC visitations, costs associated with transportation and time spent visiting healthcare facilities may inhibit women with limited resources from accessing deworming medication [47,48]. This argument may also be useful for explaining the observation that employed women were more likely to use deworming medication than their unemployed counterparts. Specifically, employed women may be more likely to have a sense of stability and financial security, possibly enabling them to prioritize their health and healthcare needs, including deworming medication use [35]. Thus, interventions to increase the uptake of deworming medication among women should target women residing in lower-income households.

Our analysis also showed that pregnant women with higher usage of ANC services were more likely to have used deworming medication during pregnancy. This is consistent with other studies that found that pregnant women who adhered to the WHO-recommended eight ANC visits were more likely to receive deworming medication as part of their care [45]. Studies in Ethiopia [49] and Tanzania [7] have revealed similar findings. Frequent visits to ANC health centers can increase pregnant women’s exposure to information about the importance of deworming medication and the potential risks of parasitic infections, which may be useful for dispelling misconceptions that can prevent them from taking these medications [7,20]. One example of an intervention that can be implemented in Benin to improve ANC services, and consequently increase the uptake of deworming medication, is a community program called “Badienou Gokh”, which has been implemented in Senegal [50]. The Badienou Gokh initiative helps improve the use of maternal and child health services by connecting pregnant women with Badienou Gokhs, or godmothers, who are women residing in the community who can mentor pregnant women about their healthcare needs and issues that may arise [50,51]. Early results stated that there has been increased use of both skilled birth attendance and antenatal consultations [50].

There are some noteworthy limitations to this study. For example, this study was based on the BDHS, which is a self-reported survey. Specifically, it is possible that some respondents had difficulty recalling pertinent questions about their deworming status, leading to the possibility of respondent bias. In addition, the BDHS is a cross-sectional survey; therefore, our results are limited to the statistical association. Finally, although our study was based on quantitative methods, qualitative studies are also helpful for explaining the contextual factors that contribute to deworming medication use. To address these issues, we recommend that future research employs longitudinal analysis, as well as in-depth qualitative analysis to unpack the processes of deworming medication use among pregnant women in Benin.

## 5. Conclusions

Our study sought to understand the factors associated with deworming medication use among pregnant women in Benin. Pregnant women are at an increased risk of contracting parasitic infections due to changes in their immune systems and hormonal levels. These infections can cause a range of health problems, including anemia, malnutrition, and low birth weight. There is an increased risk of complications during pregnancy, childbirth, and the postpartum period, as a result of parasitic infections. Particularly in Benin, there are several barriers that can impact the utilization of deworming medication for pregnant women. We found that various demographic, socioeconomic and health-related factors interplay to impact the uptake of deworming medication among pregnant women. For instance, adolescent and young pregnant women were more likely to use deworming medication compared to their older counterparts. Additionally, pregnant women who were educated, wealthier and had employment were more likely to use deworming medication compared to their counterparts. Further, women who visited ANC eight times or more were more likely to use deworming medication compared to their counterparts who visited ANC fewer than eight times. Based on these findings, we recommend a number of policies, such as community-level educational campaigns and involving community leaders, to reduce misconceptions about illnesses and medications. The recommended policies help improve access to knowledge, wealth, and healthcare services, particularly antenatal care. Implementing these policies will increase the uptake of deworming medication among pregnant women in Benin and elsewhere in SSA to improve their health and achieve SDG 3.1.

## Figures and Tables

**Table 1:** Sample characteristics.

	Percentage
**Use of deworming medication**	
No	35
Yes	65
**Age of respondents**	
15–24	24
25–34	49
35–49	27
**Marital status**	
Married	92
Not married	8
**Religion**	
Christian	50
Muslim	33
Traditionalist	10
Other	7
No religion	
**Region of residence**	
Ouémé	9
Alibori	13
Atacora	9
Atlantic	12
Borgou	13
Collines	7
Couffo	6
Donga	7
Littoral	4
Mono	4
Plateau	6
Zou	10
**Place of residence**	
Urban	39
Rural	61
**Education**	
Secondary/higher education	17
Primary education	18
No education	65
**Household wealth**	
Richest	19
Richer	21
Middle	20
Poorer	20
Poorest	20
**Employment status**	
Employed	81
Unemployed	19
**Number of ANC visits**	
Eight times or more	9
Fewer than eight times	91
**Total**	8994

**Table 2:** Logistic regression predicting the use of deworming medication in Benin.

	Unadjusted			Adjusted		
	OR	95% CI		OR	95% CI	
**Age of respondents**						
15–24	1.00			1.00		
25–34	1.02	0.90	1.15	0.90	0.80	1.03
35–49	0.76 ***	0.66	0.88	0.79 **	0.66	0.93
**Marital status**						
Married	1.00			1.00		
Not married	1.11	0.90	1.36	0.95	0.75	1.20
**Religion**						
Christian	1.00			1.00		
Muslim	0.31 ***	0.26	0.38	0.70 **	0.57	0.86
Traditionalist	0.61 ***	0.47	0.77	1.06	0.84	1.35
Other	0.63 *	0.41	0.98	0.51 **	0.31	0.83
No religion	0.29 ***	0.22	0.39	0.84	0.66	1.08
**Region of residence**						
Ouémé	1.00			1.00		
Alibori	0.06 ***	0.04	0.10	0.16 ***	0.10	0.25
Atacora	0.03 ***	0.02	0.06	0.06 ***	0.03	0.10
Atlantic	0.53 **	0.34	0.81	0.52 **	0.33	0.84
Borgou	0.03 ***	0.02	0.05	0.06 ***	0.04	0.10
Collines	0.12 ***	0.07	0.18	0.15 ***	0.09	0.24
Couffo	0.09 ***	0.06	0.14	0.10 ***	0.06	0.17
Donga	0.12 ***	0.07	0.20	0.22 ***	0.13	0.37
Littoral	0.61	0.32	1.17	0.34 **	0.17	0.68
Mono	0.17 ***	0.10	0.29	0.19 ***	0.11	0.32
Plateau	0.20 ***	0.12	0.34	0.28 ***	0.17	0.47
Zou	0.23 ***	0.14	0.37	0.25 ***	0.15	0.41
**Place of residence**						
Urban	1.00			1.00		
Rural	0.55 ***	0.45	0.68	0.89	0.72	1.10
**Education**						
Secondary/higher education	1.00			1.00		
Primary education	0.63 ***	0.52	0.77	0.61 ***	0.48	0.77
No education	0.31 ***	0.26	0.36	0.49 ***	0.40	0.59
**Household wealth**						
Richest	1.00			1.00		
Richer	0.41 ***	0.32	0.52	0.64 ***	0.49	0.83
Middle	0.25 ***	0.20	0.32	0.52 ***	0.40	0.69
Poorer	0.19 ***	0.15	0.24	0.46 ***	0.35	0.59
Poorest	0.10 ***	0.07	0.13	0.31 ***	0.23	0.41
**Employment status**						
Employed	1.00			1.00		
Unemployed	0.51 ***	0.44	0.58	0.53 ***	0.45	0.61
**Number of ANC visits**						
Eight times or more	1.00			1.00		
Fewer than eight times	0.26 ***	0.20	0.33	0.65 ***	0.50	0.85

* *p* < 0.05, ** *p* < 0.01, *** *p* < 0.001.

## Data Availability

Data for this study are available at https://dhsprogram.com/data/available-datasets.cfm.

## References

[1] World Health Organization (2022). Deworming Adolescent Girls and Women of Reproductive Age: Policy Brief. https://www.who.int/publications/i/item/9789240037670.

[2] Hailegebriel T. (2017). Prevalence of Intestinal Parasitic Infections and Associated Risk Factors among Students at Dona Berber Primary School, Bahir Dar, Ethiopia. BMC Infect. Dis..

[3] Teimouri A., Alimi R., Farsi S., Mikaeili F. (2022). Intestinal Parasitic Infections among Patients Referred to Hospitals Affiliated to Shiraz University of Medical Sciences, Southern Iran: A Retrospective Study in Pre- and Post-COVID-19 Pandemic. Environ. Sci. Pollut. Res..

[4] World Health Organization Deworming: Every Girl and Every Woman Has the Right to Be Treated. https://www.who.int/news/item/02-02-2018-deworming-every-girl-and-every-woman-has-the-right-to-be-treated.

[5] Zegeye B., Keetile M., Ahinkorah B.O., Ameyaw E.K., Seidu A.-A., Yaya S. (2021). Utilization of Deworming Medication and Its Associated Factors among Pregnant Married Women in 26 Sub-Saharan African Countries: A Multi-Country Analysis. Trop. Med. Health.

[6] De Silva N.R., Brooker S., Hotez P.J., Montresor A., Engels D., Savioli L. (2003). Soil-transmitted helminth infections: Updating the global picture. Trends Parasitol..

[7] Bankanie V., Moshi F.V. (2022). Factors Associated with the Use of Deworming Drugs during Pregnancy in Tanzania; an Analysis from the 2015–2016 Tanzanian HIV and Malaria Indicators Survey. BMC Pregnancy Childbirth.

[8] Ibikounlé M., Onzo-Aboki A., Doritchamou J., Tougoué J.-J., Boko P.M., Savassi B.S., Siko E.J., Daré A., Batcho W., Massougbodji A. (2018). Results of the First Mapping of Soil-Transmitted Helminths in Benin: Evidence of Countrywide Hookworm Predominance. PLoS Negl. Trop. Dis..

[9] United Nations Goal 3: Ensure Healthy Lives and Promote Well-Being for All at All Ages. https://sdgs.un.org/goals/goal3.

[10] Parija S.C., Chidambaram M., Mandal J. (2017). Epidemiology and clinical features of soil-transmitted helminths. Trop. Parasitol..

[11] United Nations Children’s Fund (UNICEF) and World Health Organization (2019). Progress on Household Drinking Water, Sanitation and Hygiene 2000–2017. Special Focus on Inequalities. https://www.unicef.org/nicaragua/media/1326/file/JMP%202019%20-%20complete%20layout_clean.pdf.

[12] Atabati H., Kassiri H., Shamloo E., Akbari M., Atamaleki A., Sahlabadi F., Linh N.T.T., Rostami A., Fakhri Y., Khaneghah A.M. (2020). The Association between the Lack of Safe Drinking Water and Sanitation Facilities with Intestinal *Entamoeba* Spp. Infection Risk: A Systematic Review and Meta-Analysis. PLoS ONE.

[13] Truscott J.E., Hardwick R.J., Werkman M., Saravanakumar P.K., Manuel M., Ajjampur S.S.R., Ásbjörnsdóttir K.H., Khumbo K., Witek-McManus S., Simwanza J. (2021). Forecasting the Effectiveness of the DeWorm3 Trial in Interrupting the Transmission of Soil-Transmitted Helminths in Three Study Sites in Benin, India and Malawi. Parasit. Vectors.

[14] Geyer R.E., Ibikounlé M., Emmanuel-Fabula M., Roll A., Avokpaho E., Elijan A., Wèkè L.C., Togbevi C.I., Chabi F., Houngbégnon P. (2020). Gender Norms and Mass Deworming Program Access in Comé, Benin: A Qualitative Assessment of Gender-Associated Opportunities and Challenges to Achieving High Mass Drug Administration Coverage. PLoS Negl. Trop. Dis..

[15] Bangert M., Bancalari P., Mupfasoni D., Mikhailov A., Gabrielli A.F., Montresor A. (2019). Provision of Deworming Intervention to Pregnant Women by Antenatal Services in Countries Endemic for Soil-Transmitted Helminthiasis. PLoS Negl. Trop. Dis..

[16] The Lancet Microbe (2022). Including Women in Deworming the World. Lancet Microbe.

[17] Mireku M.O., Boivin M.J., Davidson L.L., Ouédraogo S., Koura G.K., Alao M.J., Massougbodji A., Cot M., Bodeau-Livinec F. (2015). Impact of Helminth Infection during Pregnancy on Cognitive and Motor Functions of One-Year-Old Children. PLOS Negl. Trop. Dis..

[18] Boko P.M., Ibikounle M., Onzo-Aboki A., Tougoue J.-J., Sissinto Y., Batcho W., Kinde-Gazard D., Kabore A. (2016). Schistosomiasis and Soil Transmitted Helminths Distribution in Benin: A Baseline Prevalence Survey in 30 Districts. PLoS ONE.

[19] Avokpaho E.F.G.A., Houngbégnon P., Accrombessi M., Atindégla E., Yard E., Rubin Means A., Kennedy D.S., Littlewood D.T.J., Garcia A., Massougbodji A. (2021). Factors Associated with Soil-Transmitted Helminths Infection in Benin: Findings from the DeWorm3 Study. PLoS Negl. Trop. Dis..

[20] Walia B., Kmush B.L., Lane S.D., Endy T., Montresor A., Larsen D.A. (2021). Routine Deworming during Antenatal Care Decreases Risk of Neonatal Mortality and Low Birthweight: A Retrospective Cohort of Survey Data. PLOS Negl. Trop. Dis..

[21] World Health Organization Deworming Women during Pregnancy Has a Positive Effect on Child Survival and Health. https://www.who.int/news/item/29-04-2021-deworming-women-during-pregnancy-has-a-positive-effect-on-child-survival-and-health.

[22] Christian P., Khatry S.K., West K.P. (2004). Antenatal Anthelmintic Treatment, Birthweight, and Infant Survival in Rural Nepal. Lancet.

[23] Ndyomugyenyi R., Kabatereine N., Olsen A., Magnussen P. (2008). Malaria and Hookworm Infections in Relation to Haemoglobin and Serum Ferritin Levels in Pregnancy in Masindi District, Western Uganda. Trans. R. Soc. Trop. Med. Hyg..

[24] Tanou M., Kishida T., Kamiya Y. (2021). Geographical Access to Health Facilities and Maternal Healthcare Utilization in Benin: A Cross-Sectional Study. https://assets.researchsquare.com/files/rs-406731/v1/34846a00-0d83-479e-a335-2427d4301c18.pdf?c=1631881424.

[25] Agresti A., Finlay B. (2009). Statistical Methods for the Social Sciences.

[26] Gordon R. (2015). Regression Analysis for the Social Sciences.

[27] The Center for Reproductive Law and Policy (1999). Women’s Reproductive Rights in Benin: A Shadow Report.

[28] Okedo-Alex I.N., Akamike I.C., Ezeanosike O.B., Uneke C.J. (2019). Determinants of Antenatal Care Utilisation in Sub-Saharan Africa: A Systematic Review. BMJ Open.

[29] Singh L., Rai R.K., Singh P.K. (2012). Assessing the Utilization of Maternal and Child Health Care among Married Adolescent Women: Evidence from India. J. Biosoc. Sci..

[30] Mekonnen Y., Mekonnen A. (2002). Utilization of Maternal Health Care Services in Ethiopia. https://dhsprogram.com/pubs/pdf/fa38/01-mekonnen.pdf.

[31] Greenaway E.S., Leon J., Baker D.P. (2012). Understanding the Association between Maternal Education and Use of Health Services in Ghana: Exploring the Role of Health Knowledge. J. Biosoc. Sci..

[32] Adeyanju G.C., Sprengholz P., Betsch C. (2022). Understanding Drivers of Vaccine Hesitancy among Pregnant Women in Nigeria: A Longitudinal Study. npj Vaccines.

[33] Amoak D., Kye N.O., Anfaara F.W., Sano Y., Antabe R. (2023). Maternal Tetanus Toxoid Vaccination in Benin: Evidence from the Demographic and Health Survey. Vaccines.

[34] Aziato L., Odai P.N.A., Omenyo C.N. (2016). Religious Beliefs and Practices in Pregnancy and Labour: An Inductive Qualitative Study among Post-Partum Women in Ghana. BMC Pregnancy Childbirth.

[35] Zegeye B., El-khatib Z., Ameyaw E.K., Seidu A., Ahinkorah B.O., Keetile M., Yaya S. (2021). Breaking Barriers to Healthcare Access: A Multilevel Analysis of Individual- and Community-Level Factors Affecting Women’s Access to Healthcare Services in Benin. Int. J. Environ. Res. Public Health.

[36] Atuoye K.N., Amoyaw J.A., Kuuire V.Z., Kangmennaang J., Boamah S.A., Vercillo S., Antabe R., McMorris M., Luginaah I. (2017). Utilisation of Skilled Birth Attendants over Time in Nigeria and Malawi. Glob. Public Health.

[37] Ruktanonchai C.W., Ruktanonchai N.W., Nove A., Lopes S., Pezzulo C., Bosco C., Alegana V.A., Burgert C.R., Ayiko R., Charles A.S.E.K. (2016). Equality in Maternal and Newborn Health: Modelling Geographic Disparities in Utilisation of Care in Five East African Countries. PLoS ONE.

[38] Carmona A., Callahan S., Banke K. (2014). Benin Private Health Sector Census.

[39] Shewamene Z., Dune T., Smith C.A. (2017). The Use of Traditional Medicine in Maternity Care among African Women in Africa and the Diaspora: A Systematic Review. BMC Complement. Altern. Med..

[40] Sano Y., Antabe R., Atuoye K.N., Hussey L.K., Bayne J., Galaa S.Z., Mkandawire P., Luginaah I. (2016). Persistent Misconceptions about HIV Transmission among Males and Females in Malawi. BMC Int. Health Hum. Rights.

[41] Aboagye R.G., Seidu A.A., Ahinkorah B.O., Cadri A., Frimpong J.B., Hagan J.E., Kassaw N.A., Yaya S. (2022). Association between Frequency of Mass Media Exposure and Maternal Health Care Service Utilization among Women in Sub-Saharan Africa: Implications for Tailored Health Communication and Education. PLoS ONE.

[42] Tey N.-P., Lai S. (2013). Correlates of and Barriers to the Utilization of Health Services for Delivery in South Asia and Sub-Saharan Africa. Sci. World J..

[43] Aina O., Gautam L., Simkhada P., Hall S. (2020). Prevalence, Determinants and Knowledge about Herbal Medicine and Non-Hospital Utilisation in Southwest Nigeria: A Cross-Sectional Study. BMJ Open.

[44] Lipsky A.B. (2011). Evaluating the strength of faith: Potential comparative advantages of faith-based organizations providing health services in sub-Saharan Africa. Public Adm. Dev..

[45] Belay D.G., Kibret A.A., Diress M., Gela Y.Y., Sinamaw D., Simegn W., Andualem A.A., Seid A.M., Bitew D.A., Seid M.A. (2022). Deworming among Preschool Age Children in Sub-Saharan Africa: Pooled Prevalence and Multi-Level Analysis. Trop. Med. Health.

[46] Zegeye B., Ahinkorah B.O., Ameyaw E.K., Seidu A.A., Yaya S. (2021). Utilization of Deworming Drugs and Its Individual and Community Level Predictors among Pregnant Married Women in Cameroon: A Multilevel Modeling. BioMed Res. Int..

[47] Antabe R., Kansanga M., Sano Y., Kyeremeh E., Galaa Y. (2020). Utilization of Breast Cancer Screening in Kenya: What Are the Determinants?. BMC Health Serv. Res..

[48] Atuoye K.N., Dixon J., Rishworth A., Galaa S.Z., Boamah S.A., Luginaah I. (2015). Can She Make It? Transportation Barriers to Accessing Maternal and Child Health Care Services in Rural Ghana. BMC Health Serv. Res..

[49] Mengist H.M., Zewdie O., Belew A. (2017). Intestinal Helminthic Infection and Anemia among Pregnant Women Attending Ante-Natal Care (ANC) in East Wollega, Oromia, Ethiopia. BMC Res. Notes.

[50] World Health Organization (2012). Addressing the Challenge of Women’s Health in Africa Report of the Commission on Women’s Health in the African Region. https://www.afro.who.int/sites/default/files/2017-06/report-of-the-commission-on-womens-health-in-the-african-region---full-who_acreport-comp%20(1).pdf.

[51] Danailov S. (2022). Tambacounda’s Unsung Heroes, in Southeastern Senegal. UNICEF. https://www.unicef.org/senegal/en/stories/tambacoundas-unsung-heroes-southeastern-senegal.

